# An epidemiological perspective on the future of direct-to-consumer personal genome testing

**DOI:** 10.1186/2041-2223-1-10

**Published:** 2010-10-04

**Authors:** A Cecile JW Janssens, Cornelia M van Duijn

**Affiliations:** 1 Department of Epidemiology, Erasmus University Medical Center Rotterdam, the Netherlands

## Abstract

Personal genome testing is offered via the internet directly to consumers. Most tests that are currently offered use data from genome-wide scans to predict risks for multiple common diseases and traits. The utility of these tests is limited, predominantly because they lack predictive ability and clear benefits for disease prevention that are specific for genetic risk groups. In the near future, personal genome tests will likely be based on whole genome sequencing, but will these technological advances increase the utility of personal genome testing? Whole genome sequencing theoretically provides information about the risks of both monogenic and complex diseases, but the practical utility remains to be demonstrated. The utility of testing depends on the predictive ability of the test, the likelihood of actionable test results, and the options available for the reduction of risks. For monogenic diseases, the likelihood of known mutations will be extremely low in the general population and it will be a challenge to recognize new causal variants among all rare variants that are found using sequencing. For complex diseases, the predictive ability of genetic tests will be mainly restricted by the heritability of the disease, but also by the genetic complexity of the disease etiology, which determines the extent to which the heritability can be understood. Given that numerous genetic and non-genetic risk factors interact in the causation of complex diseases, the predictive ability of genetic models will likely remain modest. Personal genome testing will have minimal benefits for individual consumers unless major breakthroughs are made in the near future.

## 

An increasing number of companies are offering health-related personal genome testing via the internet directly to consumers. Over time, these products have evolved from testing a few variants for a single health outcome to testing hundreds of thousands genetic variants genome-wide for multiple outcomes simultaneously. These tests provide information about predisposition to drug response and risk predictions for a variety of diseases. For example, DeCODEme is currently predicting risks for 50 different diseases, traits and medication responses, Navigenics for 40 and 23 and Me for 66. The outcomes predicted range from various cancers, to Alzheimer disease, to Warfarin response and eye color (accessed 14 July 2010). The utility of these tests is far from clear, not in the least because the predictive ability is still limited for most diseases, and risk predictions remain subject to change as long as new variants are being discovered [1-4].

To facilitate the discovery of new variants, next-generation whole genome sequencing is increasingly utilized in genetic research. The arrays used for genome wide scans include a very large but finite number of common variants covering the genome based on the principle of linkage disequilibrium. In contrast, whole genome sequencing documents the entire genome, base pair by base pair, and thus comprises more DNA variations, such as rare variants, copy number and structural variations with potentially larger effects on clinically relevant outcomes. Whole genome sequencing will be instrumental to discover more common variants implicated in complex outcomes, but may also reveal rare causal genetic variants for monogenic diseases that are private to specific populations or even to persons. Because whole genome sequencing gives a more complete coverage, it is beyond doubt that companies will consider the technique to predict predisposition to drug response and risks of complex and monogenic diseases. Whole genome sequencing is still rather expensive, particularly to obtain the high analytic validity that is required to make predictions at the individual level, but costs are decreasing rapidly. Yet, the question is whether these technological advances will also increase the utility of personal genome testing? This will depend on whether whole genome sequencing will be able to increase the predictive ability and the expected benefits of testing.

## Predictive ability of personal genome testing

In the first place, the utility is determined by the question "will the personal genome testing have appreciable predictive ability to tell the future development of disease?" [5]. The predictive ability is evaluated for each disease separately and may differ considerably between diseases tested within the same scan, particularly between monogenic and complex traits. The difference in the predictive ability between monogenic and complex diseases is explained by two aspects of the genetic etiology, namely the heritability and the genetic complexity of the disease.

**Figure 1:**
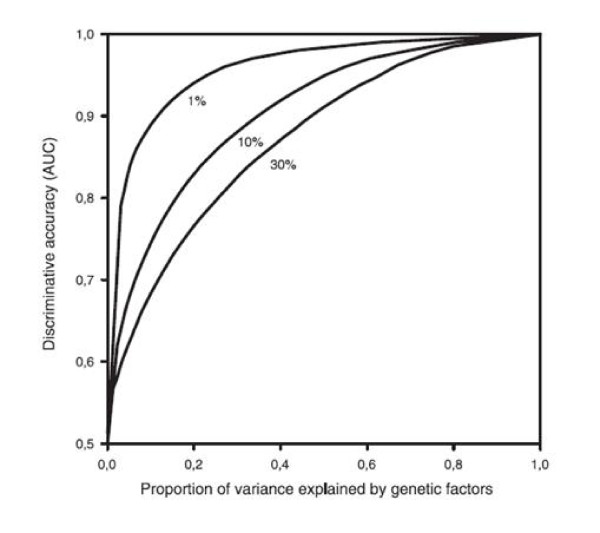
**Relationship between the proportion of variance explained by genetic factors and the maximum discriminative accuracy of genetic testing**. The discriminative accuracy, assessed as the Area Under the receiver operating characteristic Curve (AUC), is the extent to which predicted risks can discriminate between individuals who will develop the disease of interest and those who will not. The AUC is the probability that the test correctly identifies the person who will develop the disease from a pair of whom one will be affected and one will remain unaffected, and ranges from 0.5 (total lack of discrimination) to 1.0 (perfect discrimination). The numbers next to the smoothed lines refer to the risk of disease in the population. Reprinted with permission from ref 7 (Copyright 2006, *Wolters Kluwer Health*).

Monogenic diseases such as Huntington disease and cystic fibrosis are highly heritable. Although there may be multiple genetic and non-genetic factors influencing prognosis, mutations in a limited number of specific genes by themselves are sufficient causes of disease and testing the absence or presence of these mutations accurately predicts future disease development in families. Complex diseases on the other hand are caused by an intricate interplay of many genetic and non-genetic factors, such as smoking, alcohol consumption, diet and physical activity. The predictive ability of genetic risk models is determined by the combined effect of all genetic risk factors tested, and therefore indirectly by the frequency and effects of all variants included in the model. Empirical studies so far have shown that the predictive ability for most complex diseases is still moderate at best, which is for a large part explained by the relatively limited number of low-risk variants that have been discovered so far [6]. But even if all genetic variants were discovered in the future, still the predictive ability would be restricted by the fact that complex diseases are only partially heritable. Table 1 shows the wide range of heritability estimates that is observed for complex diseases and traits, ranging from 22% for happiness to >99% for eye color. Figure 1 illustrates how the heritability relates to the maximum discriminative accuracy that can be obtained when all common and rare variants that constitute the heritability are identified [7,8]. This figure shows that theoretically an almost perfect genetic test will be possible for type 1 diabetes, but that for type 2 diabetes with a heritability of 26% and an estimated disease risk between 15-25% the discriminative accuracy will remain moderate.

**Table 1:** Heritability estimates of various complex diseases and traits

Disease or trait	Heritability	Reference
Eye color	> 99%	[18]
Type 1 diabetes	88%	[19]
Schizophrenia	81%	[20]
Alzheimer's disease	79%	[21]
Height	70-87% (m), 68-85% (v)	[22]
Obesity	65-84% (m), 64-79% (w)	[23]
Smoking persistence	59% (m), 46% (w)	[24]
Anorexia nervosa	56%	[25]
Rheumatoid arthritis	53-65%	[26]
Panic disorder	43%	[27]
Prostate cancer	42%	[28]
Migraine	40-50%	[29]
Heart attack	38% (m), 57% (w)	[30]
Smoking initiation	37% (m), 55% (w)	[24]
Depression	37%	[31]
Colorectal cancer	35%	[28]
Anxiety disorder	32%	[27]
Homosexuality	30% (m), 50-60% (w)	[32]
Breast cancer	27%	[28]
Type 2 diabetes	26%	[33]
Lung cancer	26%	[28]
Happiness	22% (m), 41% (w)	[34]

Heritability and frequency estimates are obtained from published studies and meta-analyses. m = men, w = women.

Although the predictive ability of a genetic test for type 2 diabetes can become comparable to that of current non-genetic risk prediction models, this is only achieved when all genetic variants are identified. Underlying Figure 1 is the assumption that the total heritability can be explained. Whether this is a realistic assumption depends on the second major determinant: the complexity of the genetic etiology. It will be difficult to completely tease out genetic etiologies when there are many low-risk genetic variants implicated, such as the hundred that have been identified for blood cholesterol. These variants may interact with each other and with other non-genetic risk factors in many different ways. We still have little knowledge of the interactions that are anticipated from a biological perspective. So far, sophisticated genetic models that did consider interactions have not outperformed simple additive models [9,10], but these studies have most likely lacked the power to investigate even two-factor interactions. Very complex interactions involving multiple genetic and non-genetic factors have been beyond the scope statistically and computationally, because increased or decreased risks due to complex interactions are difficult to detect. When ten variants are interacting, all cases and controls in epidemiological studies will have unique *genetic profiles*, making it impossible to accurately quantify risks of disease for specific combinations of variants [6]. And when more than 20 variants are interacting, even the world population may not be large enough to determine the risks for specific genetic profiles. Unless the assumption of additive effects of genetic and non-genetic risk factors is correct, these simple examples show that the more complex the genetic etiology of a trait, the less likely it is that the genetic contribution will be fully understood in all its complexity.

**Figure 2:**
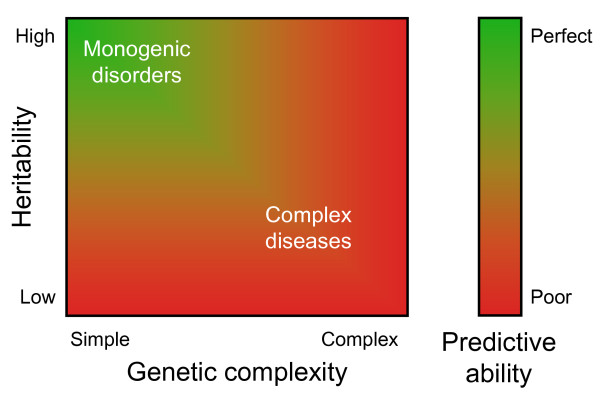
**Relationship between the heritability, genetic complexity and predictive ability of personal genome testing**.

The impact of heritability and genetic complexity on the potential predictive ability of diseases is outlined in Figure 2. When diseases are highly heritable and have a simple genetic etiology, e.g., as in monogenetic disorders, genetic testing will be accurate and very predictive. Whether these variants are mutations, haplotypes, copy number variations, insertion/deletions, or even missing or extra copies of an entire chromosome, the presence of the variants means that one will develop the disease and the absence that one will not. Also traits that are predominantly determined by one or a few variants, such as human eye color, may be predicted with relatively high accuracy [11]. When diseases are only partially influenced by genetic factors and the heritability is in the lower ranges, the predictive ability of tests that consider genetic variants only will never be very accurate when non-genetic risk factors are not included in the test. There are however examples of traits with low heritability and simple genetic etiology for which genetic testing may be clinically relevant. These include genetic effects on responses to medication, where one or a few variants, e.g., in the cytochrome P450 genes, have a large effect but do by themselves not fully determine treatment response. In contrast, when diseases are highly heritable but genetically complex, the predictive ability may not come close to the theoretical maximum, because the complexity hampers accurate assessment of the risks associated with the specific combinations of variants.

## Expected benefits of personal genome testing

The utility of personal genome testing is also determined by the expected benefits for the individual who undergoes testing. The range of potential benefits for genome wide tests is broad and includes not only enabling decisions about preventive or therapeutic interventions, but also more subjective benefits such as enhancing motivation, making plans for the future, and simply knowing one's risks of disease. These subjective benefits of testing have led to the recognition that in addition to clinical and public health utility, there is personal utility that will likely vary among consumers [12-14].

A challenge in the evaluation of the expected benefits is the fact that genome-wide scans, and in the future also whole genome sequencing, address multiple diseases at the same time and typically are not advertised and ordered for the prediction of one specific disease. This means that the benefits of testing should be evaluated across all conditions and traits that are tested, because it is not known in advance for which disease one will benefit from testing. This feature increases the utility of personal genome testing, as by definition, the probability of carrying a mutation or being at high risk for at least one disease is higher than that for one specific disorder.

However, the expected benefits of testing not only depend on what benefits may occur, but also on the likelihood of their occurrences. Even though the benefits of testing may be higher when more diseases are tested for, the likelihood of having at least some benefit may still be quantitatively low. For example, 23andme tests three mutations in BRCA1/2 of the thousands that have been documented, but which are found solely among persons from Ashkenazi Jewish descent. The probability that women with no Ashkenazi Jewish roots will learn that they have one of these mutations is virtually zero. Similarly, many mutations for other monogenic traits are specific to populations or families, implying that also individuals with a family history of disease may test negative for documented mutations. Whole genome sequencing will likely identify new mutations in these individuals, but their impact on disease risk is unknown without evaluation in other affected and non-affected relatives. Thus, testing for rare variants with strong effects has little relevance in the absence of family specific information. Predisposition to drug responses is another example where *a priori *the expected benefits of testing are low, because the probability of these benefits is conditional on developing an indication for the specific drug and the deleterious gene variants may be population specific.

But also for complex diseases, the likelihood of benefits may be disappointingly low. The presumed rationale for learning about one's risk is that elevated risks can motivate to adopt a healthier lifestyle or other preventive strategies, but genetic risk models with moderate predictive ability are not able to distinguish sizeable subgroups with substantially elevated risks. For example, in a large-population-based cohort the average risk of type 2 diabetes was 20% and the observed range of risks predicted by a model that included 18 low-risk susceptibility variants was 7 to 45% [15]. If this test were offered by companies to consumers, approximately 5% of the consumers would learn that their risks are higher than 30% but nobody that their risk are higher than 50%. Hence, consumers who would only be motivated when their genetic risks were at least 50% could have known *a priori *that this motivational benefit should not be expected from this test. To learn whether personal genome testing might motivate, consumers should first ask themselves what risk increase would make them change their behavior and then see whether the test has sufficient predictive ability to tell that risk. Because it is argued that the key to the success of DTC companies is clarity and transparency [14,16], consumers should have insight in the expected benefits and the predictive ability prior to purchasing.

## Concluding remarks

Even though most direct-to-consumer companies are still offering personal genome testing on the basis of genome-wide scans, it is clear that this technique will be outdated as soon as whole genome sequencing becomes affordable to consumers. Standard genome-wide scans will increasingly fail to cover the latest variants for the prediction of complex diseases when whole genome sequencing is used for variant discovery, and they do not cover variants for monogenic traits. Whole genome sequencing covers the entire DNA sequence, which means that all DNA variations can be read including common and rare variants implicated in monogenic and complex outcomes.

For monogenic diseases, whole genome sequencing will have the same predictive ability as tests of known mutations that are currently conducted in the clinical genetics practice. Whole genome sequencing can simultaneously test for many monogenic diseases, which increases the likelihood that individuals might benefit from knowing their DNA for at least one disease. This feature of whole genome sequencing certainly contributes to a strong sense of personal utility. However, in the absence of a positive family history or early symptoms, the *a priori *probability of carrying a known mutation will be low. At the same time, whole genome sequencing will also reveal numerous mutations with unknown impact that have never been seen before. These unknown mutations are difficult to interpret. High-throughput algorithms that combine information from biology, bioinformatics, proteomics and population genetics might be developed in the future to predict the effect of the many variants encountered, but at present one needs family, epidemiological or functional studies to quantify the deleterious effect of such mutations.

For complex diseases, whole genome sequencing will also certainly outperform prediction based on genome-wide scans, because more variants are captured. Yet, because complex diseases are only partly determined by genetic variants or are genetically too complex, it should not be expected that prediction of complex diseases becomes markedly better. When the predictive ability remains limited and prevention is effective even in the absence of a genetically increased risk, the likelihood that test results will lead to any benefit or intelligible decisions at the a personal level, is very low.

Does this mean that whole genome sequencing will have no utility at all? This is clearly not the case. There are many tests in health care that have moderate predictive ability for the individual, but these are found useful to make decisions about prevention or treatment for larger groups of people. Similarly, there will be numerous opportunities in health care in which whole genome sequencing likely may be implemented to improve health outcomes in patients and populations; opportunities where a moderate predictive ability is sufficient and the expected benefits are higher. Moderate predictive ability may be sufficient in population-based screening programs, such as breast cancer mammography screening, where whole genome sequencing could be used to differentiate in starting age or frequency of monitoring [17]. Expected benefits may be larger when tests are targeted to specific at-risk populations, e.g., to identify the genetic cause of congenital disorders in newborns, to search and subsequently test private mutations in families with a positive history of hereditary disease, or to test predisposition to specific drug responses upon indication of symptoms.

Undoubtedly, whole genome sequencing will be implemented in health care when it leads to better health outcomes for populations, even if at the personal level the benefits are modest and many individuals receive a wrong or unnecessary preventive or therapeutic intervention. Health care payers will finance predictive tests that benefit populations at large, but it is unlikely that individual consumers will massively spend money on expensive tests that have moderate predictive ability and unclear personal benefits.

## References

[B1] Mihaescu R van Hoek M Sijbrands EJ Uitterlinden AG Witteman JC Hofman A van Duijn CM Janssens AC Evaluation of risk prediction updates from commercial genome-wide scans Genet Med 2009 11 588 94 10.1097/GIM.0b013e3181b13a4f 19636253

[B2] Patch C Sequeiros J Cornel MC Direct to consumer genetic tests Eur J Hum Genet 2009 17 1111 10.1038/ejhg.2009.66 19401718 PMC2986592

[B3] Hunter DJ Khoury MJ Drazen JM Letting the genome out of the bottle--will we get our wish? N Engl J Med 2008 358 105 7 10.1056/NEJMp0708162 18184955

[B4] van Ommen GB Cornel MC Recreational genomics? Dreams and fears on genetic susceptibility screening Eur J Hum Genet 2008 16 403 4 10.1038/ejhg.2008.32 18354423

[B5] Hamburg MA Collins FS The Path to Personalized Medicine N Engl J Med 2010 363 301 4 10.1056/NEJMp1006304 20551152

[B6] Janssens ACJW Van Duijn CM Genome-based prediction of common diseases: advances and prospects Hum Mol Genet 2008 17 R166 R173 10.1093/hmg/ddn250 18852206

[B7] Janssens ACJW Aulchenko YS Elefante S Borsboom GJJM Steyerberg EW Van Duijn CM Predictive testing for complex diseases using multiple genes: fact or fiction? Genet Med 2006 8 395 400 10.1097/01.gim.0000229689.18263.f4 16845271

[B8] Wray NR Yang J Goddard ME Visscher PM The genetic interpretation of area under the ROC curve in genomic profiling PLoS Genet 2010 6 e1000864 10.1371/journal.pgen.1000864 20195508 PMC2829056

[B9] Liu F van Duijn K Vingerling JR Hofman A Uitterlinden AG Janssens AC Kayser M Eye color and the prediction of complex phenotypes from genotypes Curr Biol 2009 19 R192 R193 10.1016/j.cub.2009.01.027 19278628

[B10] Yu W Liu T Valdez R Gwinn M Khoury MJ Application of support vector machine modeling for prediction of common diseases: the case of diabetes and pre-diabetes BMC Med Inform Decis Mak 2010 10 16 10.1186/1472-6947-10-16 20307319 PMC2850872

[B11] Liu F Ikram MA Janssens AC Schuur M de Koning I Isaacs A Struchalin M Uitterlinden AG den Dunnen JT Sleegers K Bettens K Van Broeckhoven C van Swieten J Hofman A Oostra BA Aulchenko YS Breteler MM van Duijn CM A Study of the SORL1 Gene in Alzheimer's Disease and Cognitive Function J Alzheimers Dis 2009 18 51 64 19584446 10.3233/JAD-2009-1137

[B12] Grosse SD McBride CM Evans JP Khoury MJ Personal utility and genomic information: look before you leap Genet Med 2009 11 575 6 10.1097/GIM.0b013e3181af0a80 19623080 PMC3417335

[B13] Foster MW Mulvihill JJ Sharp RR Evaluating the utility of personal genomic information Genet Med 2009 11 570 4 10.1097/GIM.0b013e3181a2743e 19478683

[B14] Helgason A Stefansson K The past, present, and future of direct-to-consumer genetic tests Dialogues Clin Neurosci 2010 12 61 8 20373667 10.31887/DCNS.2010.12.1/ahelgason PMC3181949

[B15] van Hoek M Dehgan A Witteman JCM Van Duijn CM Uitterlinden AG Oostra BA Hofman A Sijbrands EJ Janssens AC Predicting type 2 diabetes based on polymorphisms from genome wide association studies: a population-based study Diabetes 2008 57 3122 8 10.2337/db08-0425 18694974 PMC2570410

[B16] Khoury MJ McBride CM Schully SD Ioannidis JP Feero WG Janssens AC The Scientific Foundation for personal genomics: recommendations from a National Institutes of Health-Centers for Disease Control and Prevention multidisciplinary workshop Genet Med 2009 11 559 67 10.1097/GIM.0b013e3181b13a6c 19617843 PMC2936269

[B17] Pharoah PD Antoniou AC Easton DF Ponder BA Polygenes. Polygenes, risk prediction, and targeted prevention of breast cancer N Engl J Med 2008 358 2796 803 10.1056/NEJMsa0708739 18579814

[B18] Zhu G Evans DM Duffy DL Montgomery GW Medland SE Gillespie NA Ewen KR Jewell M Liew YW Hayward NK Sturm RA Trent JM Martin NG A genome scan for eye color in 502 twin families: most variation is due to a QTL on chromosome 15q Twin Res 2004 7 197 210 10.1375/136905204323016186 15169604

[B19] Hyttinen V Kaprio J Kinnunen L Koskenvuo M Tuomilehto J Genetic liability of type 1 diabetes and the onset age among 22,650 young Finnish twin pairs: a nationwide follow-up study Diabetes 2003 52 1052 5 10.2337/diabetes.52.4.1052 12663480

[B20] Sullivan PF Kendler KS Neale MC Schizophrenia as a complex trait: evidence from a meta-analysis of twin studies Arch Gen Psychiatry 2003 60 1187 92 10.1001/archpsyc.60.12.1187 14662550

[B21] Gatz M Reynolds CA Fratiglioni L Johansson B Mortimer JA Berg S Fiske A Pedersen NL Role of genes and environments for explaining Alzheimer disease Arch Gen Psychiatry 2006 63 168 74 10.1001/archpsyc.63.2.168 16461860

[B22] Silventoinen K Sammalisto S Perola M Boomsma DI Cornes BK Davis C Dunkel L De Lange M Harris JR Hjelmborg JV Luciano M Martin NG Mortensen J Nisticò L Pedersen NL Skytthe A Spector TD Stazi MA Willemsen G Kaprio J Heritability of adult body height: a comparative study of twin cohorts in eight countries Twin Res 2003 6 399 408 10.1375/136905203770326402 14624724

[B23] Schousboe K Willemsen G Kyvik KO Mortensen J Boomsma DI Cornes BK Davis CJ Fagnani C Hjelmborg J Kaprio J De Lange M Luciano M Martin NG Pedersen N Pietiläinen KH Rissanen A Saarni S Sørensen TI Van Baal GC Harris JR Sex differences in heritability of BMI: a comparative study of results from twin studies in eight countries Twin Res 2003 6 409 21 10.1375/136905203770326411 14624725

[B24] Li MD Cheng R Ma JZ Swan GE A meta-analysis of estimated genetic and environmental effects on smoking behavior in male and female adult twins Addiction 2003 98 23 31 10.1046/j.1360-0443.2003.00295.x 12492752

[B25] Bulik CM Sullivan PF Tozzi F Furberg H Lichtenstein P Pedersen NL Prevalence, heritability, and prospective risk factors for anorexia nervosa Arch Gen Psychiatry 2006 63 305 12 10.1001/archpsyc.63.3.305 16520436

[B26] MacGregor AJ Snieder H Rigby AS Koskenvuo M Kaprio J Aho K Silman AJ Characterizing the quantitative genetic contribution to rheumatoid arthritis using data from twins Arthritis Rheum 2000 43 30 7 10.1002/1529-0131(200001)43:1<30::AID-ANR5>3.0.CO;2-B 10643697

[B27] Hettema JM Neale MC Kendler KS A review and meta-analysis of the genetic epidemiology of anxiety disorders Am J Psychiatry 2001 158 1568 78 10.1176/appi.ajp.158.10.1568 11578982

[B28] Lichtenstein P Holm NV Verkasalo PK Iliadou A Kaprio J Koskenvuo M Pukkala E Skytthe A Hemminki K Environmental and heritable factors in the causation of cancer--analyses of cohorts of twins from Sweden, Denmark, and Finland N Engl J Med 2000 343 78 85 10.1056/NEJM200007133430201 10891514

[B29] Ligthart L Boomsma DI Martin NG Stubbe JH Nyholt DR Migraine with aura and migraine without aura are not distinct entities: further evidence from a large Dutch population study Twin Res Hum Genet 2006 9 54 63 10.1375/twin.9.1.54 16611468

[B30] Zdravkovic S Wienke A Pedersen NL Marenberg ME Yashin AI De Faire U Heritability of death from coronary heart disease: a 36-year follow-up of 20 966 Swedish twins J Intern Med 2002 252 247 54 10.1046/j.1365-2796.2002.01029.x 12270005

[B31] Sullivan PF Neale MC Kendler KS Genetic epidemiology of major depression: review and meta-analysis Am J Psychiatry 2000 157 1552 62 10.1176/appi.ajp.157.10.1552 11007705

[B32] Kirk KM Bailey JM Dunne MP Martin NG Measurement models for sexual orientation in a community twin sample Behav Genet 2000 30 345 56 10.1023/A:1026557719181 11206089

[B33] Poulsen P Kyvik KO Vaag A Beck-Nielsen H Heritability of type II (non-insulin-dependent) diabetes mellitus and abnormal glucose tolerance--a population-based twin study Diabetologia 1999 42 139 45 10.1007/s001250051131 10064092

[B34] Bartels M Saviouk V de Moor MH Willemsen G van Beijsterveldt TC Hottenga JJ de Geus EJ Boomsma DI Heritability and genome-wide linkage scan of subjective happiness Twin Res Hum Genet 2010 13 135 42 10.1375/twin.13.2.135 20397744

